# Hospital Staff Shortage after the 2011 Triple Disaster in Fukushima, Japan-An Earthquake, Tsunamis, and Nuclear Power Plant Accident: A Case of the Soso District

**DOI:** 10.1371/journal.pone.0164952

**Published:** 2016-10-27

**Authors:** Sae Ochi, Masaharu Tsubokura, Shigeaki Kato, Shuichi Iwamoto, Shinichi Ogata, Tomohiro Morita, Arinobu Hori, Tomoyoshi Oikawa, Antoku Kikuchi, Zenjiro Watanabe, Yukio Kanazawa, Hiromi Kumakawa, Yoshinobu Kuma, Tetsuo Kumakura, Yoshimitsu Inomata, Masahiro Kami, Ryuzaburo Shineha, Yasutoshi Saito

**Affiliations:** 1 Department of Internal Medicine, Soma Central Hospital, Fukushima, Japan; 2 Department of Radiation Protection, Soma Central Hospital, Fukushima, Japan; 3 Department of General Internal Medicine, Graduate School of Biomedical and Health Sciences, Hiroshima University, Hiroshima, Japan; 4 Soso Public Health Institute, Fukushima, Japan; 5 Department of Psychology, Hibarigaoka Hospital, Fukushima, Japan; 6 Minamisoma Municipal General Hospital, Fukushima, Japan; 7 Onoda Hospital, Fukushima, Japan; 8 Kashima Kosei Hospital, Fukushima, Japan; 9 Public Soma General Hospital, Fukushima, Japan; 10 Department of Urology, Public Soma General Hospital, Fukushima, Japan; 11 Ohmachi Hospital, Fukushima, Japan; 12 Institute of Medical Science, University of Tokyo, Tokyo, Japan; 13 Soma Central Hospital, Fukushima, Japan; 14 Hirata Central Hospital, Fukushima, Japan; King Abdullah International Medical Research Center, SAUDI ARABIA

## Abstract

**Introduction:**

In 2011, Fukushima was struck by a triple disaster: an earthquake, tsunamis, and a nuclear accident. In the aftermath, there was much fear among hospital staff members about radiation exposure and many staff members failed to report to work.

**Objectives:**

One objective is to measure this shortage in hospital staff and another is to compare the difference in recovery by hospital types and by categories of hospital staff.

**Design:**

The monthly records of the number of staff members from May 2011 to September 2012 were extracted anonymously from the records of 7 local hospitals in the Soso district in Fukushima. Change in the number of staff was analyzed.

**Results:**

Staff shortages at hospitals reached a maximum within one month after the disaster (47% reported to work). The shortage of clerks was the most severe (38% reported to work), followed by nurses (48% reported to work). The shortages remained even 18 months after the disaster.

**Conclusion:**

After a disaster in which the damage to hospital functions surpasses the structural damage, massive support of human resources in the acute phase and a smaller volume of support in the mid-term phase appear to be required, particularly for non-medical staff.

## Introduction

Hospitals are vital assets for local communities to maintain the health of their populations. After a huge disaster, the need for healthcare in affected areas greatly increases due to the injuries associated with damage from the disaster and deterioration of chronic medical conditions in the short term, as well as the aftermath of the disaster in the long term. In this regard, enhancing the capabilities of local hospitals beforehand to manage such unusual situations is one of the highest priorities of disaster mitigation because disasters may endanger the functions of hospitals in many ways. For example, after meteorological or geographical disasters such as floods, earthquakes, and landslides, the structural and infrastructural damage to hospitals has been reported to impair hospital functions [1]. In addition to such natural disasters, chemical, biological, radiological, and nuclear (CBRN) disasters are also known to hinder hospital functions at various levels. For instance, fear of invisible hazards that hampered the willingness to work in the affected areas has been observed among hospital staff members, resulting in hospital malfunction due to staff shortages [2].

On March 11, 2011, the second largest nuclear accident in history occurred at the Fukushima Daiichi nuclear power plant (NPP) in Fukushima Prefecture, Japan, following an unprecedented earthquake and subsequent tsunamis. This triple disaster—an earthquake, tsunamis, and an NPP accident—severely affected the area around the plant.

**Fig 1:**
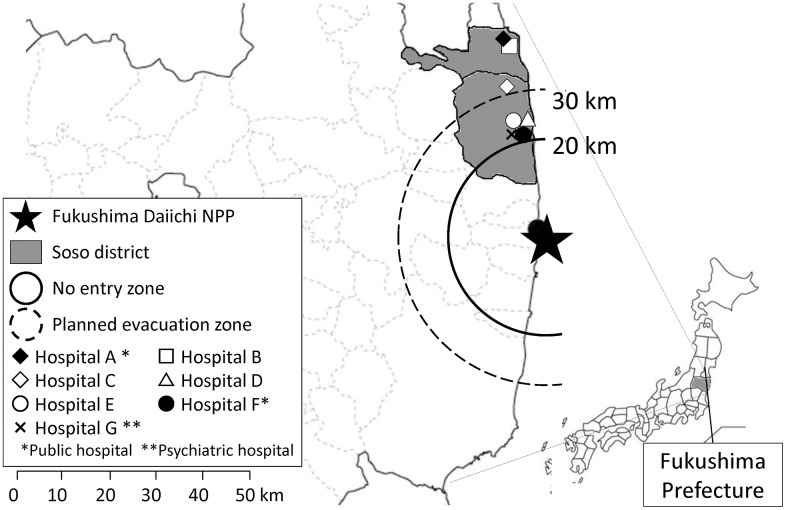
Locations of Soso district and participated hospitals.

One of the affected areas, the Soso district, is located approximately 15–40 km north of the plant and was one of the areas most affected by this triple disaster (Fig 1). Two of the twelve hospitals located in this area experienced catastrophic damage due to the tsunamis and ceased to function immediately after the disaster. Although the physical damage to the other hospitals was not as severe, fear among the hospital staff about radiation emitted from the NPP resulted in much of the staff failing to report to work as soon as further hydrogen detonation of the damaged nuclear reactor at the plant was reported on television [3]. Soso district had suffered from shortage in medical staff even before the disaster. The number of medical doctors in 2010, a year before the disaster, was 120.4 per 100,000 residents, which is nearly a half of the average of whole Japan (219.0 per 100,000 residents) [4]. The number of nurses was also quite less (579.1 per 100,000 residents), when compared with the national average in 2008 was 980 per 100,000 residents [5]. Thus, it could be assumed that the entire capacity of the hospital function in this area had been already compromised significantly due to severe shortages in the staffs before the disaster. However, information about how many hospital staff members left their workplace after the triple disaster, and how long it took to recover the required number of staff is still lacking. To address this issue, the present study was undertaken by surveying the changes in the number of hospital staff during the 18 months after the triple disaster in the Soso district and targeted health professionals and medical clerks. The findings illustrate the length of time needed to recover from staff shortages at hospitals and the types of hospitals that experienced the most severe shortages, thereby providing data for future planning when dispatching hospital staff after disasters.

## Materials and Methods

### The situation

On March 11, 2011, an earthquake of magnitude 9.0 and subsequent tsunamis of up to 14 m struck the northeast coast of Japan. The tsunamis disabled the cooling system of the reactors of the NPP. Meltdown started soon after the tsunamis, and the first visible explosion, which was suspected to be caused by leaked hydrogen gas, occurred on March 12. Over the following 3 weeks, several suspected hydrogen detonations occurred.

In response to the nuclear accident, the Japanese National Government established the following evacuation zones on March 12, 2011: a compulsory no-entry zone within a 20-km radius of the NPP, in which all of the residents were mandated to evacuate; and a planned evacuation zone within a 20- to 30-km radius from the NPP, in which the residents were recommended to stay at home to avoid excessive external radiation exposure. Although evacuation from the planned evacuation zones was voluntary, mass evacuation occurred in this area due to fear of radiation. As a result, the population of the Soso district was reported to decrease from nearly 100,000 to 40,000 after the evacuation order [6].

### Studied hospitals

Twelve hospitals were in the Soso district before the disaster. Three hospitals were located within no-entry zones, and all of the hospitalized patients were transferred to other hospitals by March 14. Two other hospitals sustained catastrophic damage from the disaster, and stopped functioning on March 11. Therefore, this survey was conducted on the remaining seven hospitals. Informed consent was obtained from each hospital prior to beginning the survey.

### Data collection

The monthly records of the number of staff members from May 2011 to September 2012 were extracted anonymously from the records of each hospital. The number of outpatient visits during the same period were also collected. Staff members were categorized as medical doctors, nurses, other clinical staff (OCS), and hospital clerks. OCS included pharmacists, clinical laboratory technicians, clinical engineers, radiology technicians, physiotherapists, occupational therapists, dieticians, and nurse’s aides.

Following the rules of the Soso Public Health Institute, the number of part-time staff members was converted into a number of full-time workers by dividing their weekly working hours by those of full-time workers.

Monthly data on number of resistered residents in Soso district was also calculated based using municipal data [7, 8].

### Ethical considerations

Written informed consent was obtained from every hospital. The institutional review board of the University of Tokyo approved this study.

## Results

All seven hospitals agreed to participate in the survey. Fig 1 and Table 1 show the location and characteristics of the hospitals, respectively. All are small-to-medium sized hospitals with 80–240 beds and 90.9–259.0 employees (full-time worker equivalents) before the disaster. Distances from the NPP ranged from 24.4 km (Hospital E) to 44.5 km (Hospital A). Two hospitals (Hospitals A and F) are public hospitals, whereas the others are privately owned. Hospital G specializes in psychiatric care.

**Table 1:** Characteristics of and number of staff members at Soso district hospitals (Source from Soso Public Health Institute).

	Founder	Care Type	Number of beds	Distance from the NPP (km)	Number of staff members before the disaster				Total
					Doctor	Nurse	OCS	Clerk	
A	Public	General	240	44.5	28.1	143.8	26	57	254.9
B	Private	General	97	43.8	8.2	48.3	46	26	128.5
C	Private	General	80	32.6	6.1	37.8	12	35	90.9
D	Private	General	188	25.0	14.4	97.5	17	48	176.8
E	Private	General	199	24.5	8.6	78.0	5	108	199.6
F	Public	General	230	24.4	20.5	132.5	40	66	259.0
G	Private	Psychiatric	254	24.4	5.7	74.0	50	33	162.7
**Total**			**1288**		**91.6**	**611.8**	**196**	**373**	**1272.4**

NPP: nuclear power plant; OCS: other clinical staff

### Decrease in the number of staff members immediately after the disaster

**Fig 2:**
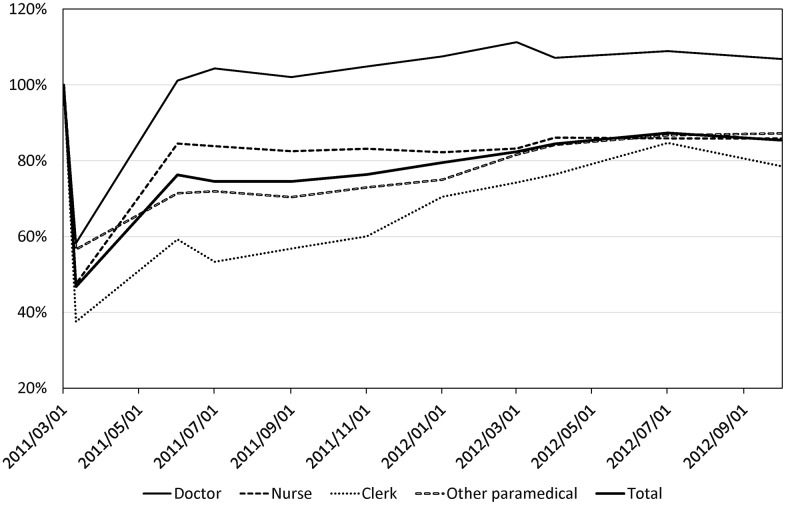
The % change in the number of hospital staff members working in the Soso district during the 18 months after the triple disaster.

The fluctuation of the number of staff, patients, and resistered residents in Soso district was shown in S1 Table. The time trend of the percentage change in the number of hospital staff members compared to the number before the disaster (May 1, 2011) is shown in Fig 2. The number fell sharply soon after the disaster. On April 1, 2011 (20 days after the first hydrogen detonation), the total number of staff members was 595 (47% reported to work). The percentages of staff members who reported to work showed that among all of the occupational categories, the shortage in the number of clerks was the most severe (38% reported to work), followed by nurses (48% reported to work), OCS (57% reported to work), and medical doctors (58% reported to work).

### Recovery of the number of staff and patients

Three months after the disaster, the number of staff members started to recover gradually, but had not fully recovered even at 18 months after the disaster. In September 2012, the recovery rate remained at 85% (1,087). The recovery was slowest in clerks (79% compared to that on March 1, 2011) followed by nurses (85%) and OCS (87%). Interestingly, the number of medical doctors increased by 6% compared to that before the disaster (Fig 2).

### Burden of hospital staff

**Fig 3:**
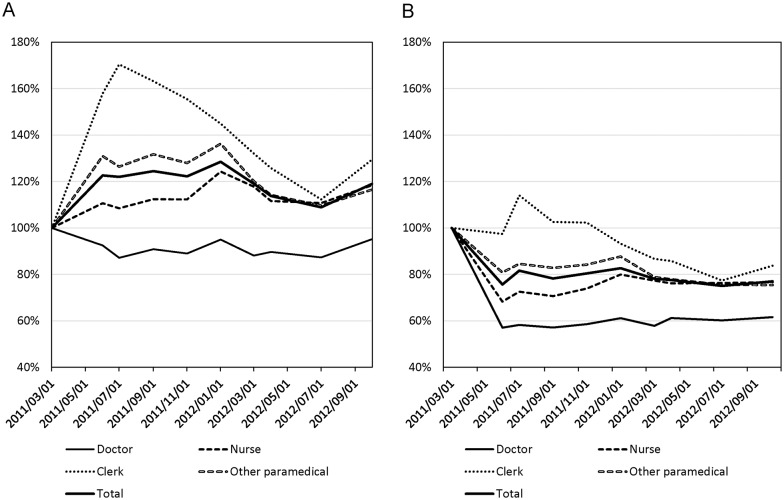
The % change in the estimated workload of staff members. The numbers of outpatients (A) and inpatients (B) were divided by the number of staff members.

In the Soso district, the number of residents also declined sharply after the disaster. Although registered residents in Soso district decreased only by 10% during 18 months after the disaster (S1 Table), actual decrease would be much larger than this because many evacuees left their registration. Therefore the decrease in the number of hospital staff members might be proportional to that of the number of patients. To address this point, we calculated the number of outpatients and inpatients per hospital staff member, which was used as a tentative index of the staff workload (Fig 3A and 3B). Outpatients per staff increased except among medical doctors, which reached a maximum several months after the disaster (Fig 3A). Among nurses, the maximum was 124% in January 2012 (10 months after the disaster), the maximum among OCS was 131% in September 2011 (6 months after the disaster), and the maximum among clerks was 170% in July 2011 (4 months after the disaster). Even at 18 months after the disaster, the overall staff workload remained increased by 18%.

On the other hand, inpatients per staff decreased except clerks (Fig 3B). This decrease was caused mainly owing to limitation of the hospitals to accept inpatients due to shortage in staff. For example, Hospital F closed a floor due to lack of nurses. Hospital G was forced to evacuate psychiatric patients, since they needed long-term hospitalization. Therefore, Fig 3A rather than Fig 3B is presumed to represent actual burden to hospital staff.

### Differences between hospitals

**Fig 4:**
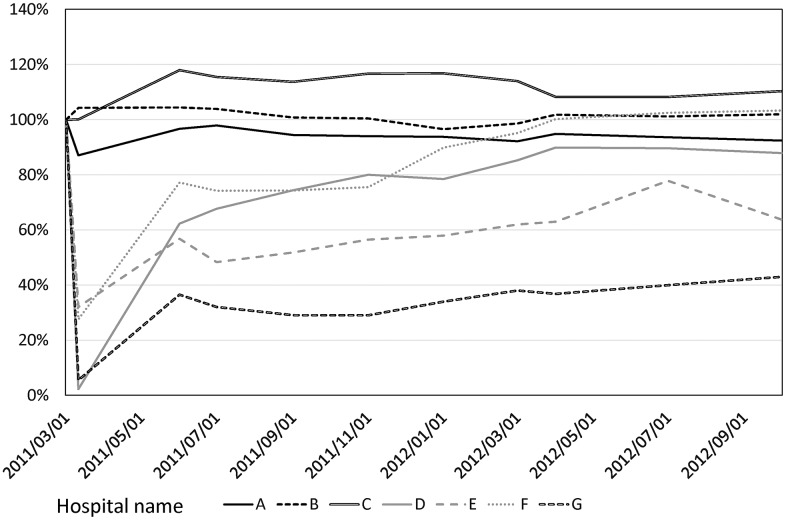
The % change in the number of hospital staff members at each hospital within 18 months after the triple disaster.

The extent of staff reduction and speed of recovery varied among the different hospitals (Fig 4). The shortage of hospital staff in the acute phase was apparent among those located within 25 km of the NPP (Hospitals D, E, F, and G). Two of the seven hospitals (Hospitals D and G) temporarily closed with no staff. Both of these hospitals are private and are located within 25 km of the NPP. For these two hospitals, the recovery rates after 18 months were much lower than those of the other hospitals. In particular, Hospital G, which specializes in psychiatric care, had a very low recovery rate of 43%. In contrast, another hospital (Hospital F), which is a public general hospital located within 25 km of the NPP, experienced almost full recovery with respect to the number of staff members within 18 months of the disaster.

## Discussion

This is the first study to illustrate how the number of hospital staff members fluctuates after a huge disaster. Staff shortages at hospitals reached a maximum within 1 month after the disaster but recovered to some extent after 3 months. Even so, the shortages remained even 18 months after the disaster. This study also revealed that the speed of recovery varied by the category of hospital staff.

### Complex reasons of unwillingness

Sharp decreases in the healthcare staff during the acute phase of a disaster, as observed in the Soso district, have also been observed at other nuclear accident sites. After the nuclear accident at Three Mile Island, only 7 of more than 70 medical doctors reported for service at a hospital [9].

Several reasons may explain the unwillingness of staff to work after a disaster. First, a psychological fear may exist among the staff. The closer the houses of the staff members are to the NPP, the stronger the fear and anxiety about radiation exposure might be. Therefore, staff living near the plant are more likely to evacuate. Second, disruption of the supply of commodities such as food, fuel, and the means of transportation. Many distributors outside of the Soso district refused to send their staff within a 30-km radius of the NPP based on ground security [10]. As a result, these officially habitable areas adjacent to the no-entry zone were confronted with a shortage of basic necessities such as food and fuel, which caused mass evacuation by the residents. Third, many staff members, especially mothers with small children, were anxious about radiation exposure to their children rather than that to themselves, and voluntarily decided to evacuate [10]. This situation may partly explain why the number of nurses and clerks, majority of whom were women, decreased more rapidly than that of medical doctors. Fourth, after evacuation there was a situation for the residents evacuated from the affected areas to feel guilty of leaving from their patients and colleagues. Such situation was exampled by a case in the other area of Fukushima in which a medical doctor was told by her supervisor: ‘If you abandon your patients and run away, you cannot return to this hospital forever. You’ll be ostracized.’ [11] Such stigmatization sometimes is still making it difficult for those who had evacuated to come back to their own workplaces in the affected areas.

### Possible measures to prevent staff from leaving

There is no report on effective intervention to prevent staff from leaving their workplace during such social disruption. However, based on the aforementioned background, at least the following conditions might be needed to prevent hospital staff from taking unprepared evacuation.

First, approach to provide timely information about the source of fear needs to be established. In Soso district, little information was available on the outcomes of the NPP accidents, which fostered fear among the staff at local medical services [10]. Second, assurance of security for not only hospital staff but also their family members is needed. For example, this could be achieved by preparedness of personal protection equipment for family members. Moreover, offer of job opportunities to their partners and educational opportunities for their children might be important to retain staff who prioritize their family members.

Third, minimizing the disruption of the supply chain of essential life resources such as food, water, and fuel is indispensable to retain hospital staffs to continue work at the affected areas after a huge disaster because hospital staffs were unable to stay at their workplace without external resources. On top of these, it is essential to build up atmosphere where no other staff member is blamed for the leavers in their evacuating, so that the evacuated staffs will feel it easy to return to their workplace without hesitation.

Whether financial support has some effect on retaining staff is still an open question, but it is essential to retain function of local hospitals. In Soso district, Tokyo Electric Power Co., Inc., the owner of the Fukushima Daiichi Nuclear Power Plant, compensated the damage of the hospital based on yearly income of each hospital. Prefectural government of Fukushima also subsidize the hospitals to reconstruction of their buildings. Most of the hospitals, especially private hospitals, might not be able to continue care without these supports.

### Difference in recovery speed

#### Difference by job types

Interestingly, the number of medical doctors rather increased after the disaster, owing simply not to return of the evacuated medical doctors. Rather, the disaster area was attractive in newly recruiting many young medical doctors, who were willing to conduct their clinical and academic practices related with disaster medicine or public health research. For example, before disaster junior residents out of Fukushima prefecture did not join Minamisoma Municipal General Hospital. However, during 5 years after the disaster, 11 junior residents as well as many more senior residents from the nation-wide areas have already joined in Minamisoma Municipal General for medical cares, and many of young doctors have been also scientifically very active by writing academic papers and case reports related to the disaster. [12, 13].

On the contrary, only few nurses and OCS have come from the outside to date. To retain nurses and OCS, further research is needed to identify factors that made the staff remain at the hospitals.

#### Difference by hospitals

The speed in recovery varied also among the hospitals. Psychiatric hospitals are often more severely affected by any damage by disasters [14], presumably due to less support from outside. Furthermore, this study revealed that hospitals B and C, private hospitals, could retain a number of staffs immediately after the disaster, while hospital A, a public hospital, experienced a decrease in the staff number even though it located at the farthest from the NPP. Apparent increase of the staff numbers of hospital B and C was due to acceptance of the other hospital staff evacuated from no-entry zone (within 20 km radius from the NPP). Therefore, although many staff members at hospitals B and C evacuated, total number of the staffs during those periods overtly increased. On the contrary, as hospital A, a public hospital, it was still mandatory to take legal employment procedures to accept such staff from the other hospitals, thus were not permitted to accept the evacuated staff in acute phase. We thus presume that these facts are very suggestive for future plans for quick staff mobilization need to be established for coming disasters.

### Lessons learned for external support in disaster settings

The fact that the number of hospital staff members decreased by up to 50% strongly suggests that the number of rescue teams dispatched to the disaster area was not sufficient to support the functions of local healthcare systems. After the Great East Japan Earthquake, approximately 380 disaster medicine assistance teams (DMATs) with 1,800 members entered the disaster area, including Fukushima [15]. However, given the staff shortage in the Soso district alone, the human resource requirements appear to have far exceeded the supply.

The results of this study also suggest that sufficient human resource support is important not only for maintaining hospital functions but also for achieving early recovery. Two hospitals that closed their outpatient wards (Hospitals E and G) still suffered from low recovery rates even at 18 months, whereas a hospital that retained its functions during the acute phase (Hospital F) experienced a faster recovery than other hospitals.

#### Short-term support

Intensive short-term human resources support from external sources might be important for avoiding delay in recovery of hospital functions, and such support is presumed to include not only DMATs with enhanced volume, but also supports by other hospital staffs such as medical clerks and other paramedics. In addition, such supports need to be equally distributed to both public and private hospitals.

#### Long-term support

Although the number of hospital staff members recovered to some extent within 3 months, the shortage lasted for over 1 year (Fig 2). Therefore, longer-term human resource supplies, albeit a smaller volume than that required during the acute phase, are needed, particularly after disasters that cause great social disturbance. Future plans for mitigating injuries due to nuclear disasters require a discussion of dispatching long-term medical support teams to the disaster area.

#### Range of human resource

Another issue to be addressed is the discrepancy in the speed of recovery between different categories of staff members. In particular, the recovery of the number of medical clerks was the slowest among all occupations surveyed. This result is compatible with those of a previous report suggesting that non-professional workers have a lower willingness-to-report in disaster settings [16]. Because hospitals do not function without non-clinical workers, such as cleaners, dieticians, and distributors, further study is needed to determine which types of staff members are required to maintain hospital functions, how much of a reduction in number is expected in disaster situations, and how to compensate for the lack of these workers.

### Applicability to other disasters

In addition to nuclear disasters, other CBRN disasters can also cause social disturbance due to fear [17]. For example, during the Ebola outbreak in 2014, routine healthcare systems collapsed during the outbreak due to the shunning of clinics by both patients and providers because of fear of infection [18]. In such disasters, damage to human resources due to psychological fear may exceed the physical damage to buildings. By presenting a better understanding of the numbers and types of human resources that are needed to maintain the functions of community hospitals after such a disaster, the findings of the current study are informative for establishing effective preparedness of hospitals in the face of the hazards of CBRN disasters.

### Limitations

This research was unable to provide a reason some hospitals recovered more rapidly than others. For example, it is not clear whether Hospital F recovered more quickly because it is a public hospital or because it did not close outpatient wards in the acute phase. In addition, the present assessment did not verify changes in the size of the population surrounding the hospitals, so the numbers may not fully represent the workload of each hospital. Further research may reveal more factors that influence the number of staff members at hospitals during disasters.

## Conclusions

This study describes the changes in the number of hospital staff members after a triple disaster that consisted of an earthquake, tsunamis, and a nuclear accident. Based on the present findings, massive support of human resources in the acute phase and a smaller volume of support in the mid-term phase appear to be required, particularly for non-medical staff. According to the Sendai Framework for Disaster Risk Reduction, understanding the disaster risk and the need “to systematically evaluate, record, and publicly account for disaster losses” is one of the highest priorities of action [19]. By shedding light on hospital staff losses, the results of the present study should contribute to the implementation of the Sendai framework and to global disaster risk reduction.

## Supporting Information

S1 Table Number of staff, patients, and registered residents in Soso district. (DOCX)
