# Supplementary material for: Hospital Staff Shortage after the 2011 Triple Disaster in Fukushima, Japan-An Earthquake, Tsunamis, and Nuclear Power Plant Accident: A Case of the Soso District
Source: PLoS One. 2016 Oct 27;11(10):e0164952. doi: 10.1371/journal.pone.0164952 (PMC5082811; doi:10.1371/journal.pone.0164952)
Supplement: S1 Table — (DOCX) [file pone.0164952.s001.docx]

**S1 Table.** Number of staff, patients, and registered residents in Soso district

| Date | Doctor | Nurse | Clerk | OCS | Total | Patient number | | Registered  population |
| --- | --- | --- | --- | --- | --- | --- | --- | --- |
|  |  |  |  |  |  | Outpatient | Inpatient |  |
| 2011/03/1 | 92 | 612 | 373 | 196 | 1272 | 1589 | 1010 | 109548 |
| March 2011  (After disaster) | 53 | 291 | 140 | 111 | 595 | N.A | N.A | 108044 |
| 2011/06/01 | 93 | 5117 | 221 | 140 | 971 | 1623 | 616 | 105826 |
| 2011/07/01 | 96 | 513 | 199 | 141 | 949 | 1578 | 595 | 105191 |
| 2011/09/01 | 94 | 505 | 212 | 138 | 948 | 1610 | 605 | 104542 |
| 2011/11/01 | 96 | 509 | 224 | 143 | 972 | 1620 | 658 | 104187 |
| 2012/01/01 | 99 | 503 | 263 | 147 | 1012 | 1773 | 603 | 103878 |
| 2012/03/01 | 102 | 509 | 277 | 160 | 1048 | 1701 | 681 | 103576 |
| 2012/05/01 | 98 | 527 | 285 | 165 | 1075 | 1668 | 649 | 102864 |
| 2012/07/01 | 100 | 526 | 316 | 170 | 1111 | 1650 | 655 | 102560 |
| 2012/10/01 | 98 | 525 | 293 | 171 | 1087 | 1764 | 640 | 102598 |
